# Maternal Opioid Drug Use during Pregnancy and Its Impact on Perinatal Morbidity, Mortality, and the Costs of Medical Care in the United States

**DOI:** 10.1155/2014/906723

**Published:** 2014-08-28

**Authors:** Valerie E. Whiteman, Jason L. Salemi, Mulubrhan F. Mogos, Mary Ashley Cain, Muktar H. Aliyu, Hamisu M. Salihu

**Affiliations:** ^1^Division of Maternal-Fetal Medicine, Department of Obstetrics and Gynecology, College of Medicine, University of South Florida, 2 Tampa General Circle, 6th Floor, Tampa, FL 33606, USA; ^2^Maternal and Child Health Comparative Effectiveness Research Group, Department of Epidemiology and Biostatistics, College of Public Health, University of South Florida, 13201 Bruce B. Downs Boulevard MDC 56, Tampa, FL 33612, USA; ^3^Department of Community and Health Systems, School of Nursing, University of Indiana, 1111 Middle Drive, Indianapolis, IN 46202, USA; ^4^Department of Health Policy and Medicine, Vanderbilt University, 2525 West End Avenue, Suite 750, Nashville, TN 32703, USA

## Abstract

*Objective*. To identify factors associated with opioid use during pregnancy and to compare perinatal morbidity, mortality, and healthcare costs between opioid users and nonusers. *Methods*. We conducted a cross-sectional analysis of pregnancy-related discharges from 1998 to 2009 using the largest publicly available all-payer inpatient database in the United States. We scanned ICD-9-CM codes for opioid use and perinatal outcomes. Costs of care were estimated from hospital charges. Survey logistic regression was used to assess the association between maternal opioid use and each outcome; generalized linear modeling was used to compare hospitalization costs by opioid use status. *Results*. Women who used opioids during pregnancy experienced higher rates of depression, anxiety, and chronic medical conditions. After adjusting for confounders, opioid use was associated with increased odds of threatened preterm labor, early onset delivery, poor fetal growth, and stillbirth. Users were four times as likely to have a prolonged hospital stay and were almost four times more likely to die before discharge. The mean per-hospitalization cost of a woman who used opioids during pregnancy was $5,616 (95% CI: $5,166–$6,067), compared to $4,084 (95% CI: $4,002–$4,166) for nonusers. *Conclusion*. Opioid use during pregnancy is associated with adverse perinatal outcomes and increased healthcare costs.

## 1. Introduction

Opioid pain medications are among the most prescribed drugs in the United States (US) [1]. In the past few decades, recent trends in increases in narcotic abuse overshadow successes in improved awareness and management of pain. Clinicians, administrators, and policymakers now face the consequential task of preventing opioid drug misuse and addiction without compromising their effective and appropriate use in the treatment of pain.

Opioid dependence in pregnancy complicates the clinical management of an already vulnerable group of patients. Dependence increases the risk of poor maternal and perinatal outcomes [2–11]. Women of reproductive age who use and abuse opioid drugs, both prescription and illegal, are more likely to have a lower socioeconomic status, family instability, receive inadequate prenatal care, and suffer from alcohol, tobacco, and illicit drug use [12, 13]. In addition to the risks associated with opioid dependence, these comorbid conditions further increase the risk of adverse perinatal outcomes [3, 14].

Increasing at an alarming rate, opioid use in pregnancy underwent an estimated 3-4-fold increase between 2000 and 2009 [15, 16]. The 2011 National Survey on Drug Use and Health reports of the United States found 5% of pregnant women 15 to 44 years of age report using illicit drugs [17]. These data suggest an urgent need to evaluate, on a national level, not only the negative health outcomes associated with maternal opioid use during pregnancy, but also the related economic cost burden on the US healthcare system. In this study, we leveraged a large, nationally representative hospital discharge database to identify factors that are associated with an increased likelihood of opioid use during pregnancy. We then compared selected maternal and fetal outcomes between opioid users. Finally, we computed the direct inpatient medical costs associated with maternal opioid use.

## 2. Materials and Methods

A cross-sectional analysis of all pregnancy-related hospital discharges from 1998 to 2009 was conducted using the National Inpatient Sample (NIS), the largest publicly available all-payer inpatient database in the US, made available by the Healthcare and Cost Utilization Project (HCUP) [18]. To create the dataset each year, HCUP stratifies all nonfederal community hospitals from participating states by five major hospital characteristics: rural/urban location, number of beds, geographic region, teaching status, and ownership. A random sample of 20% of hospitals from each stratum is then drawn using a systematic random sampling technique [18].

Hospital discharges for women who were pregnant or delivered were identified using the variable “NEOMAT” in the NIS dataset. This indicator was created by HCUP to identify maternal and/or neonatal diagnosis records on the basis of International Classification of Diseases, Ninth Revision, Clinical Modifications (ICD-9-CM) diagnosis and procedure codes for pregnancy and delivery [19]. After identifying the study population, we scanned ICD-9-CM codes (principal and secondary) in each woman's discharge record for an indication of opioid use during pregnancy, as well as for selected maternal/fetal clinical outcomes. The complete list of ICD-9-CM codes used to characterize each condition is presented in the appendix.

Individual-level sociodemographic factors were extracted from the NIS databases. Maternal age in years was classified into five categories: <20, 20–24, 25–29, 30–34, and ≥35. Self-reported maternal race-ethnicity was first based on ethnicity (Hispanic or non-Hispanic [NH]), and the NH group further subdivided by race (white, black, or other). Relative median household income (in quartiles) was estimated by HCUP using the patient's zip code and served as a proxy for each woman's socioeconomic status. We grouped the primary payer for hospital admission into three categories: government (Medicare/Medicaid), private (commercial carriers, private health maintenance organization [HMOs], and preferred provider organization [PPOs]), and other sources (e.g., self-pay and charity). We also considered several hospital characteristics including teaching status (teaching, or a ratio of full-time equivalent interns and residents to nonnursing home beds ≥0.25, versus nonteaching), location (urban versus rural), and US region (Northeast, Midwest, South, or West).

The NIS databases also contain discharge-level charges for all hospitalized patients. Reported charges, however, are not a good estimate of actual cost, since there is significant variation in markup from what it costs a hospital to provide medical care to what it charges for services rendered [20]. To adjust for variation in markup across hospitals and over time, we multiplied the total charge for a hospital stay by a time- and hospital-specific cost-to-charge ratio (CCR) available from HCUP [21]. However, even within the same facility, sizeable differences in markup exist across different departments (e.g., higher markup for operating room services compared to routine bed services). Therefore, we incorporated into the computation of cost an “adjustment factor” (AF) that attempts to account for this intradepartmental variation to yield a more accurate cost estimate for each discharge record [22, 23]. Consider

(1) $$\begin{array}{c} \text{cost}=\left(\text{reported}\;\text{charges}*\text{CCR}*\text{AF}\right). \end{array}$$

### 2.1. Statistical Analysis

We calculated descriptive statistics including frequencies, percentages, and rates to describe prevalence of opioid use during pregnancy across maternal age, racial/ethnic, household income strata, primary payer, selected behavioral characteristics, and comorbidities. National rate estimates were computed by weighting the analyses with discharge-level weights provided in the NIS databases. We constructed simple and multivariable survey logistic regression models (SURVEYLOGISTIC procedure) to identify factors associated with an increased likelihood of maternal opioid use during pregnancy. Since use of the NIS databases confers a large sample size and a limited number of available covariates, the multivariable model included all factors considered in bivariate analyses. In addition to identifying predictors for maternal opioid use, we also calculated the rate of selected clinical comorbidities by maternal opioid use status. These comorbidities were selected based on a review of the literature and expert opinion and identified using ICD-9-CM codes in the discharge record. The comorbidities included anxiety, chronic renal disease, depression, HIV status, insomnia, obesity, osteopenia, and prepregnancy diabetes and hypertension.

We compared the rate of selected maternal/fetal pregnancy outcomes between opioid users versus nonusers. We used unconditional survey logistic regression to generate the odds ratios (ORs) and 95% confidence intervals (CIs) for outcomes associated with maternal opioid use. In addition to an unadjusted model, we constructed two multivariable models. In the first multivariable model, we adjusted for sociodemographic, perinatal, and hospital characteristics. In the second multivariable model, we also adjusted for tobacco, alcohol, and drug use, as well as existing medical conditions (obesity, chronic renal failure, prepregnancy diabetes, and prepregnancy hypertension) that may be related to both maternal opioid use and the selected pregnancy outcomes.

After computing the direct inpatient medical costs for each pregnancy-related discharge, we compared mean maternal direct hospitalization costs by opioid use status. Considering the strong positive skewness of the cost data, we used a multivariable generalized linear model with a gamma distribution and a natural log link to estimate the mean difference in cost, after adjusting for potential confounders.

All statistical analyses accounted for the complex sampling design of the NIS. To account for NIS sampling design changes, we used the NIS-Trends files, supplied by HCUP, so that trend weights and data elements would be consistently defined over time [24]. Statistical tests were two-sided with level of significance set at 5%. In addition, for cost analyses, we reweighted all discharges to account for missing cost data by multiplying the original discharge weight provided by HCUP by the ratio of the summed weights across all discharges to the summed weight of discharges with nonmissing cost information. Since hospital-level CCR data were only available beginning in 2001, we restricted cost analyses to discharges for the period 2001–09. Analyses were performed using SAS software, version 9.3 (SAS Institute, Inc., Cary, MC) and Stata statistical software, release 11 (Stata Corp LP, College Station, TX). The Institutional Review Board of the University of South Florida determined that this study using deidentified, publicly available data did not meet the definition of human subjects research and was thus exempt from IRB approval.

## 3. Results

Of the estimated 55,781,965 pregnancy-related hospitalizations, 138,224 were associated with opioid use, a prevalence of 2.5 cases per 1,000 discharges (95% CI: 2.2–2.8). The rate of opioid use during pregnancy initially decreased from 2.5 per 1,000 in 1998 to 1.6 per 1,000 in 2001. This decreasing trend was followed by a 12% annual increase to a peak rate of 4.0 per 1,000 in 2009.

The rate of opioid use during pregnancy varied considerably across maternal sociodemographic, perinatal, behavioral, and hospital characteristics (Table 1). The highest crude rates (per 1,000 discharges) were seen among women using/abusing alcohol (81.0), women using tobacco during pregnancy (21.6), women on Medicare/Medicaid (4.5) or “Other” insurance (4.8), women in the lowest quartile of household income (3.6), and black-NH women (3.2). Conversely, low rates of opioid use during pregnancy were observed among teenage mothers (0.9), women on private insurance (0.7), women of other-NH race/ethnicity (1.0), and women in the highest quartile of household income (1.4). After adjusting for other covariates, compared to women with private insurance, women without private insurance had 9 times the odds of opioid use (Medicare/Medicaid; OR = 8.9; 95% CI: 7.7–10.3; “Other” insurance; OR = 9.2; 95% CI: 8.2–10.4). Alcohol and tobacco users were approximately 7 times as likely to use/abuse opioids, relative to nonusers. The likelihood of opioid use during pregnancy increased with increasing maternal age and decreased with increasing household income (Table 1). Although black-NH women had the highest crude rates of opioid use, after adjustment for other sociodemographic and behavioral characteristics, black-NH women were significantly less likely than white-NH women to use opioids (OR = 0.6, 95% CI: 0.5–0.8). Similar findings were obtained in Hispanic and other-NH women when compared to their white-NH counterparts.

**Figure 1:**
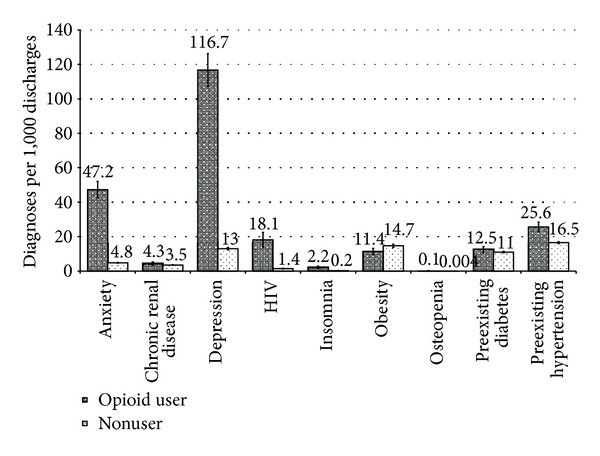
Rates (per 1,000 pregnancy-related discharges) of selected comorbidities, by opioid use status, among pregnancy-related discharges, NIS, 1998–2009. HIV = human immunodeficiency virus, NIS = Nationwide Inpatient Sample

Figure 1 presents the rates of various comorbidities, by opioid use status. Compared to nonusers, women who used opioids during pregnancy had significantly higher rates of depression (per 1,000 discharges) (116.7 versus 13.0), anxiety (47.2 versus 4.8), HIV (18.1 versus 1.4), and insomnia (2.2 versus 0.2). Users were also more likely to have chronic medical conditions such as hypertension, diabetes, and renal disease but had a slightly lower rate of medically diagnosed obesity (11.4 versus 14.7).

Maternal opioid use during pregnancy was also associated with pregnancy-related maternal/fetal morbidity and mortality (Table 2). Even after adjusting for sociodemographic, behavioral, and chronic prepregnancy conditions, opioid use was associated with an increased odds of threatened preterm labor (OR = 1.3, 95% CI: 1.2–1.5), early onset delivery (OR = 1.7, 95% CI: 1.6–1.9), poor fetal growth (OR = 1.6, 95% CI: 1.5–1.8), and stillbirth (OR = 1.3, 95% CI: 1.2–1.5). Women who used/abused opioids during pregnancy were also 4 times as likely to have a prolonged hospital stay exceeding 5 days (95% CI: 3.4–4.7) and were almost 4 times as likely to die during their hospital stay (OR = 3.7, 95% CI: 2.3–5.9). These differences in morbidity and mortality also translated into differences in direct inpatient medical costs. The mean per-hospitalization cost of a woman who used opioids during pregnancy was $5,616 (95% CI: $5,166–$6,067), compared to $4,084 (95% CI: $4,002–$4,166) for nonusers. The estimated per-hospitalization difference in cost between opioid and non-opioid-related discharges was $2,602 (95% CI: $1,931–$3,272) after adjustment for potential confounders. With an estimated 138,224 pregnancy-related hospital discharges affected by opioid use nationally from 1998 to 2009, the excess direct inpatient medical cost associated with opioid use was estimated at $359 million, or approximately $30 million annually.

## 4. Discussion

Consistent with previous studies, this multiyear population-based study found an increasing prevalence of opioids use/abuse among pregnant women in the US [1, 3–5, 7, 10, 17, 25, 26]. In our previous investigation of national trends of opioid use among pregnant mothers in the US, we discuss the alarming overall increase, as well as geographic, regional, and sociodemographic differences in both the rate and trends of opioid use over 12 years [16]. Due to concern for adverse effects on the mother and developing fetus, opioid abuse in pregnancy continues to be a major source of concern [1, 5–7, 13, 14]. As rates of opioid abuse in pregnancy are increasing the incidence of neonatal abstinence syndrome is rising [15]. This syndrome leads to prolonged neonatal hospitalizations, which in turn increase overall hospital costs among these mothers and their infants.

The current study builds on the existing literature by looking at the impact of opioid use and abuse during pregnancy on a wide range of maternal and infant birth outcomes. We found that pregnant women who used or abused opioids during pregnancy were more likely to have other comorbidities, including depression, anxiety, insomnia, diabetes, hypertension, renal diseases, and HIV infection. This finding was expected due to the association between these comorbid conditions and the development of chronic pain, poor response to pain medications, or experiencing the clinical condition during opioid withdrawal [26–28]. In our study, pregnant women who used or abused opioids were also more likely than nonusers to have a prolonged hospital stay; develop acute renal failure; and suffer mortality prior to hospital discharge. Their infants suffered from increased rates of growth restriction and stillbirth. Our findings were consistent with previous reports [29–31]. The source of worsening perinatal outcomes is likely multifactorial.

Opioid dependent women are more likely to have multiple comorbidities including mental health disorders such as depression and anxiety [32, 33]. Studies also associate maternal anxiety during pregnancy with poor neurological development in the fetus [34]. In addition to mental health disorders, opioid dependent women are more often from socially disadvantaged backgrounds [35], lack healthy nutritional habits, have inadequate prenatal care, and engage in risky sexual practices [36]. These comorbid conditions may explain the adverse infant birth outcomes observed in our study. However, the association between opioid abuse during pregnancy and adverse maternal and infant birth outcomes persisted even after controlling for potential confounders. In addition to associated comorbidities, women with opioid abuse may have irregular menses leading to unintended pregnancy [37, 38]. Adverse perinatal outcomes occur at higher rates among unintended pregnancies alone [39]. Despite possible confounding due to associated comorbidities, the increase in hospital costs and adverse perinatal outcomes in the current study is likely due to neonatal abstinence syndrome and preterm deliveries as was seen in prior investigations [7, 15, 31, 40–42]. Due to a lack of sufficiently detailed data, this hypothesis could not be tested in our study.

This strength of the current study includes the large sample size and length of study. We used an extremely large multiyear hospital discharge database that enabled us to investigate a range of maternal/fetal outcomes. To our knowledge, this is the first study to report the impact of opioid dependence during pregnancy on maternal/fetal outcomes using over a decade of nationally representative data. We also looked at the distribution of comorbidities by maternal opioid dependence status to provide clinicians and other healthcare providers a broader understanding of the complexities surrounding opioid use/abuse during pregnancy.

Despite the noted strengths, our results need to be considered in light of the following limitations. First, the analyses were based on a database of inpatient hospitalizations and, therefore, cases of maternal opioid use not captured during pregnancy-related inpatient admissions were not included in this study. The missed cases are likely limited to home births which occurs in <1% of the US population [43]. Second, our operational definition of opioid use during pregnancy relied exclusively on ICD-9-CM codes documented in the NIS databases. These diagnostic codes lack the specificity to distinguish between use and abuse of prescription versus prescribed opioids. Thus, our analyses were not able to* directly* address the increasing concern for overprescription of opioids. Third, the deidentified nature of the publicly available NIS datasets do not permit linkage of maternal delivery and infant birth hospitalizations. Therefore, we were only able to investigate a small number of fetal outcomes and could not assess birth-related events available in the infant's birth record. Third, due to the lack of a unique patient identifier, we were unable to link hospitalizations for the same woman over time and may count the same woman more than once over time leading to an overestimation of opioid use in pregnancy. Finally, hospital discharge summaries have suboptimal sensitivity to capture all instances of maternal opioid use and may lead to underreporting. However, when compared to self-reports of substance abuse, discharge data contain greater objectivity [44]. Finally, our cost analyses were conducted from a third-party payer perspective and were only able to estimate direct medical care costs from the institutional portion of the hospital stay [45]. The NIS does not contain information on physician costs or indirect costs (e.g., lost wages).

In summary, we found increased rates of maternal comorbidities, prolonged hospital stays, in-hospital mortality, and poor fetal growth and survival among women who used or abused opioids during their pregnancy. These adverse pregnancy outcomes translated into significantly increased direct costs of inpatient care. The information provided in this study will be critical to the development and implementation of appropriate services for this high-risk group of women.

## Figures and Tables

**Table 1:** Rates^a^  of opioid use by maternal sociodemographic, perinatal, behavioral, and hospital characteristics, among pregnancy-related discharges, NIS, 1998–2009.

Characteristic	*N* ^b^	Rate^a^ of opioid use (95% CI)	OR^c^ (95% CI)	AOR^d^ (95% CI)
Overall	55,781,965	2.48 (2.18–2.78)	n/a	n/a
Maternal age (years)				
13–19	6,198,796	0.93 (0.82–1.04)	0.35 (0.31–0.38)	0.23 (0.21–0.26)
20–24	13,822,023	2.47 (2.16–2.77)	0.91 (0.85–0.98)	0.62 (0.58–0.66)
25–29	15,001,057	2.70 (2.35–3.05)	Reference	Reference
30–34	12,797,406	2.76 (2.37–3.15)	1.02 (0.94–1.11)	1.36 (1.26–1.47)
≥35	7,921,464	2.84 (2.41–3.28)	1.05 (0.97–1.15)	1.37 (1.25–1.50)
Other/unknown	41,218	0.37 (<0.00–0.89)	0.14 (0.03–0.56)	0.15 (0.03–0.66)
Maternal race				
White, non-Hispanic	22,047,327	2.90 (2.52–3.28)	Reference	Reference
Black, non-Hispanic	6,025,913	3.24 (2.32–4.17)	1.12 (0.88–1.43)	0.61 (0.48–0.78)
Hispanic	9,444,177	1.33 (1.07–1.59)	0.46 (0.37–0.56)	0.31 (0.25–0.38)
Other, non-Hispanic	4,079,383	1.02 (0.80–1.24)	0.35 (0.28–0.44)	0.26 (0.21–0.34)
Missing/unknown	14,185,165	2.68 (2.02–3.35)	0.93 (0.70–1.22)	0.98 (0.78–1.23)
Tobacco use				
Yes	2,009,092	21.60 (18.71–24.50)	12.50 (11.41–13.70)	6.67 (6.07–7.33)
No	53,772,873	1.76 (1.56–1.97)	Reference	Reference
Alcohol use				
Yes	96,629	81.02 (71.00–91.04)	37.56 (33.98–41.52)	7.17 (6.42–7.99)
No	55,685,336	2.34 (2.06–2.62)	Reference	Reference
Hospital region				
Northeast	9,516,239	4.47 (3.66–5.28)	2.60 (1.88–3.60)	2.16 (1.60–2.92)
Midwest	12,032,900	2.35 (1.74–2.96)	1.37 (0.94–1.98)	0.88 (0.60–1.28)
South	20,796,015	2.13 (1.61–2.65)	1.24 (0.86–1.78)	0.87 (0.63–1.18)
West	13,436,811	1.72 (1.26–2.19)	Reference	Reference
Hospital location				
Rural	6,877,937	1.36 (1.15–1.58)	Reference	Reference
Urban	48,763,827	2.64 (2.30–2.98)	1.94 (1.58–2.38)	2.22 (1.80–2.74)
Hospital teaching status				
Nonteaching	29,501,465	1.63 (1.43–1.82)	Reference	Reference
Teaching	26,140,299	3.44 (2.85–4.04)	2.12 (1.72–2.62)	1.68 (1.33–2.12)
Hospital bed size				
Small	6,100,955	2.39 (1.84–2.94)	0.89 (0.63–1.28)	0.82 (0.59–1.13)
Medium	14,821,822	2.67 (1.94–3.39)	Reference	Reference
Large	34,718,987	2.42 (2.06–2.77)	0.91 (0.66–1.23)	0.95 (0.72–1.26)
Household income				
Lowest quartile	14,617,169	3.58 (3.06–4.10)	2.53 (2.19–2.93)	1.54 (1.36–1.82)
2nd quartile	14,070,584	2.50 (2.18–2.82)	1.76 (1.58–1.97)	1.20 (1.09–1.33)
3rd quartile	13,294,169	2.18 (1.89–2.47)	1.54 (1.41–1.67)	1.17 (1.08–1.26)
Highest quartile	12,830,659	1.42 (1.23–1.60)	Reference	Reference
Missing/unknown	969,383	3.63 (3.02–4.24)	2.56 (2.17–3.03)	1.43 (1.17–1.75)
Primary payer				
Medicare/medicaid	22,249,834	4.51 (3.89–5.14)	6.90 (6.07–7.84)	8.87 (7.66–10.27)
Private	29,701,613	0.66 (0.59–0.73)	Reference	Reference
Other	3,830,518	4.79 (4.18–5.41)	7.33 (6.54–8.22)	9.20 (8.16–10.38)

AOR = adjusted odds ratio, CI = confidence interval, NIS = Nationwide Inpatient Sample, OR = odds ratio.

^a^Per 1,000 pregnancy-related discharges.

^b^Weighted to estimate national frequency and may not add to total due to missing data.

^c^Crude model comparing the odds of maternal opioid use across different levels of each characteristic, separately.

^d^A single multivariable model includes all characteristics that appear in the table.

**Table 2:** Rates^a^  of selected clinical outcomes by opioid use status and odds ratios and 95% confidence intervals for the association between opioid use and each outcome among pregnancy-related discharges, NIS, 1998–2009.

Outcomes	Rate^a^ of outcome		OR (95% CI)		
	Opioid users	Nonopioid users	Model 1^b^	Model 2^c^	Model 3^d^
Maternal					
** **Threatened preterm labor	30.1	22.3	1.36 (1.24–1.49)	1.34 (1.22–1.47)	1.32 (1.19–1.45)
** **Early onset delivery	124.0	65.2	2.03 (1.88–2.20)	1.92 (1.77–2.07)	1.72 (1.59–1.85)
** **PROM	38.5	35.4	1.10 (1.00–1.20)	1.12 (1.03–1.23)	1.06 (0.98–1.16)
** **Wound infection	7.0	5.0	1.41 (1.18–1.68)	1.19 (1.00–1.42)	1.17 (0.98–1.40)
** **Acute renal failure	2.1	0.5	4.10 (3.11–5.41)	2.78 (2.09–3.72)	2.84 (2.11–3.84)
** **Postpartum depression^f^	24.7	2.1	12.04 (10.83–13.40)	2.09 (1.79–2.44)	1.75 (1.49–2.05)
** **Hospital stay >5 days^e^	133.4	29.9	5.00 (4.16–6.02)	4.83 (4.10–5.69)	4.02 (3.41–4.74)
** **In-hospital maternal mortality	0.8	0.1	5.89 (3.74–9.28)	3.63 (2.32–5.68)	3.69 (2.32–5.87)
Fetal					
** **Poor fetal growth	35.9	15.9	2.31 (2.10–2.55)	2.21 (2.00–2.44)	1.61 (1.46–1.77)
** **Stillbirth	10.0	6.3	1.60 (1.39–1.83)	1.41 (1.23–1.62)	1.32 (1.15–1.51)

CI = confidence interval, NIS = Nationwide Inpatient Sample, OR = odds ratio, PROM = premature rupture of membranes.

^a^Per 1,000 pregnancy-related discharges.

^b^Crude model with maternal opioid use as the only independent variable.

^c^Model 1 + adjustment for maternal age, household income, multiple birth, primary payer, and rural/urban status.

^d^Model 2 + adjustment for tobacco, alcohol, maternal obesity, chronic renal failure, diabetes mellitus, and existing hypertension.

^e^Model also adjusts for disposition at discharge.

^f^Model also adjusts for history of depression.

**Table 3:** List of International Classification of Diseases, Ninth Edition, Clinical Modification Codes used to identify selected perinatal conditions.

Condition	International Classification of Diseases, 9th Edition, Diagnosis Code
Exposure	
Opioid use	304.0x, 304.7, 305.5, 965.00, 965.01, E850.0, E935.0
Comorbidities	
Anxiety	300.0x, 309x, 293.84
Chronic renal disease	581x, 582x, 583x, 585x, 587x, 646.2x
Depression	296.2x, 296.3x, 298.0, 300.4, 301.12, 309.0, 309.1, 311
HIV	042, V08, 795.3
Insomnia	780.51, 780.52, 307.42, 327.0x, 327.15, 307.41
Obesity	278.00, 278.01, 278.03, 649.1x, V85.3x, V85.4x, V85.54, 793.91
Osteopenia	733.90
Prepregnancy diabetes	249x, 250x, 648.0x
Prepregnancy hypertension	401x, 402x, 403x, 404x, 405x, 642.0x, 642.1x, 642.2x, 642.7x
Perinatal outcomes	
Threatened preterm labor	644.0x
Early onset delivery	644.2x
Premature rupture of membranes	658.1x
Wound infection	674.1x, 674.3x, 998.3x, 998.5x
Acute renal failure	584x, 669.3x
Postpartum depression	648.40, 648.42, 648.44
Poor fetal growth	656.5x
Stillbirth	656.4x, V27.1, V27.3, V27.4, V27.6, V27.7

*The code suffix “x” represents all possible codes that follow the stated code prefix.

^†^Procedure codes, no diagnostic codes were used to define Cesarean section.

## Appendix

See Table 3.
